# The West Antarctic Ice Sheet may not be vulnerable to marine ice cliff instability during the 21st century

**DOI:** 10.1126/sciadv.ado7794

**Published:** 2024-08-21

**Authors:** Mathieu Morlighem, Daniel Goldberg, Jowan M. Barnes, Jeremy N. Bassis, Douglas I. Benn, Anna J. Crawford, G. Hilmar Gudmundsson, Hélène Seroussi

**Affiliations:** ^1^ Department of Earth Sciences, Dartmouth College, Hanover, NH 03755, USA.; ^2^ School of Geosciences, University of Edinburgh, Edinburgh, UK.; ^3^ School of Geography and Environmental Sciences, Northumbria University, Newcastle upon Tyne, UK.; ^4^ Department of Climate and Space Sciences and Engineering, The University of Michigan, Ann Arbor, MI 48109, USA.; ^5^ School of Geography and Sustainable Development, University of St Andrews, St Andrews KY16 9AL, UK.; ^6^ Division of Biological and Environmental Sciences, University of Stirling, Stirling FK9 4LA, UK.; ^7^ Thayer School of Engineering, Dartmouth College, Hanover, NH 03755, USA.

## Abstract

The collapse of ice shelves could expose tall ice cliffs at ice sheet margins. The marine ice cliff instability (MICI) is a hypothesis that predicts that, if these cliffs are tall enough, ice may fail structurally leading to self-sustained retreat. To date, projections that include MICI have been performed with a single model based on a simple parameterization. Here, we implement a physically motivated parameterization in three ice sheet models and simulate the response of the Amundsen Sea Embayment after a hypothetical collapse of floating ice. All models show that Thwaites Glacier would not retreat further in the 21st century. In another set of simulations, we force the grounding line to retreat into Thwaites’ deeper basin to expose a taller cliff. In these simulations, rapid thinning and velocity increase reduce the calving rate, stabilizing the cliff. These experiments show that Thwaites may be less vulnerable to MICI than previously thought, and model projections that include this process should be re-evaluated.

## Teaser

The most extreme projections of ice loss from the West Antarctic Ice Sheet are less likely than previously thought.

## INTRODUCTION

Among all sources of uncertainty in future sea level rise, the dynamic response of the Greenland and Antarctic ice sheets remains the largest contributor. In its latest assessment, the Intergovernmental Panel on Climate Change (IPCC) added a high-end scenario that includes a “low-likelihood, high-impact storyline” (*1*). This scenario predicts twice as much global mean sea level rise by 2100 compared to all other projections. Sea level rise under this scenario would exceed 15 m by 2300, three times more than other projections, due to the collapse of the West Antarctic Ice Sheet and parts of the East Antarctic Ice Sheet. The mass loss is more extreme than others considered because it includes the potential for a runaway process known as marine ice cliff instability (MICI).

According to the MICI hypothesis, tall and steep ice cliffs could be exposed if the floating ice shelves that fringe ice sheets collapse rapidly through a process such as hydrofracture. Above a threshold height, stresses at the cliff exceed the shear strength of ice, causing structural failure of ice and rapid retreat through calving (*2*). This process could become self-sustaining if exposed cliffs remain above the threshold height (*3*, *4*) but could be halted if changes in ice geometry reduce the terminal cliff below the threshold (*5*). Sea level projections that include MICI have so far been based on a single ice sheet model, using a fairly coarse resolution and a simple parameterization implemented as a vertical “wastage” term (*3*, *4*). This simple parameterization is based on cliff height derived from limited contemporary observations of Sermeq Kujalleq (Jakobshavn Isbræ) in Greenland and Crane Glacier in the Antarctic Peninsula (*3*, *4*). In this parameterization, cliff failure initiates when cliff heights exceed 80 m above sea level, reaching a maximum calving rate of 10 m/day for ice cliffs that exceed 100 m above sea level. On the basis of this parameterization, it has been suggested that the Antarctic Ice Sheet alone would contribute up to 1-m sea-level rise equivalent (SLE) by 2100 and exceed 15-m SLE by 2300 under high-emission scenarios (RCP 8.5) (*4*). In an updated assessment from the same authors, these projections were revised to about 0.35-m SLE by 2100 and 9-m SLE by 2300 under the same high-emission scenario (*6*).

Currently, MICI is still not widely accepted or implemented in ice sheet models because it has yet to be directly observed. Given the potential for large rates of sea level rise, if MICI is initiated, there is an urgent need to further investigate cliff failure and if/how it could become a self-sustaining process. Recently, a high-fidelity 3D model was used to investigate the conditions under which an ice cliff would fail, and the resulting calving rates from the cliff (*7*). The modeling workflow simulated the viscous deformation and brittle failure of synthetic glacier domains through a one-way coupling of the fullStokes continuum model, Elmer/Ice (viscous deformation), and the Helsinki Discrete Element Model (brittle failure). They found that, in a conservative estimate of cliff stability, cliffs could be stable up to a height of 135 m above sea level, i.e., 55 m higher than in the original parameterization (*4*). The calving rates for these cliffs would be around 1 m/day, i.e., one order of magnitude smaller than the original parameterization. However, in this revised parameterization, cliff failure increases rapidly as a function of cliff height [i.e., in the form of a power law (*7*)] instead of reaching a maximum calving rate as was previously proposed (*3*, *4*). In this parameterization, cliff failure would therefore initiate at higher cliff heights than in previous modeling work (*4*), but calving rates could largely exceed the maximum calving rate previously assumed (*4*).

To re-evaluate the vulnerability of the West Antarctic Ice Sheet to cliff failure and its potential for MICI under this revised parameterization, in this study, we model the future evolution of the Amundsen Sea Embayment following a hypothetical complete collapse of its ice shelves. We use three different ice sheet models (ISSM, STREAMICE, and Úa) that rely on different initialization methods, different mesh resolutions, and different ways of treating calving numerically (see the Materials and Methods). The goal is to ensure that our results are independently robust across different ice flow models with different numerical implementations.

## RESULTS

### Impact of ice shelf collapse

Although all three ice sheet models considered in this study include the entire Amundsen Sea Embayment, we focus the discussion on Thwaites Glacier, which is prone to rapid grounding-line retreat and high contribution to sea level rise over the 21st century (*8*). Its ice shelf is currently undergoing disintegration (*9*–*11*) and prior studies have concluded that this glacier is susceptible to rapid MICI-driven retreat (*4*).

**Fig. 1.**
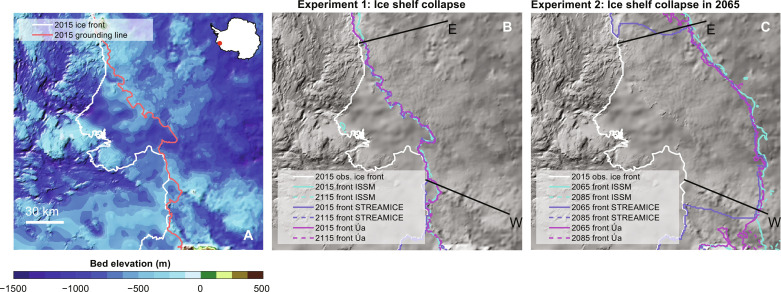
Thwaites’ bed topography and modeled ice front positions for our two collapse experiments. (**A**) Bed topography under and ocean bathymetry next to Thwaites Glacier, the white line indicates the 2015 ice front, and the orange line shows the 2015 grounding line. (**B**) Initial prescribed ice front position following the 2015 ice shelf collapse and after 100 years of simulations overlaid on bed topography. The “E” and “W” black lines indicate the extent of the cliff height sections shown in Fig. 3. (**C**) Initial prescribed ice front position after 50 years of grounding-line retreat and 20 years beyond.

**Fig. 2.**
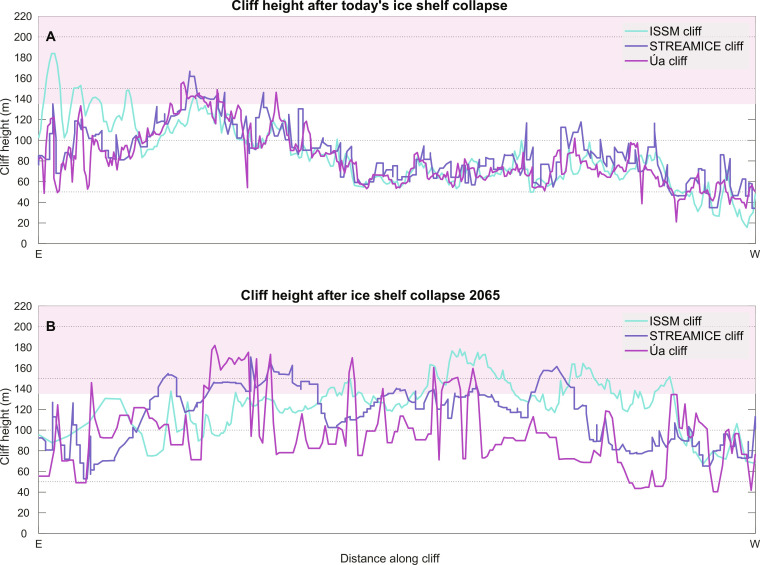
Cliff heights of the three models after a catastrophic collapse. (**A**) The cliff heights along the black lines crossing the eastern and western sections of the Thwaites Glacier terminus, as shown in Fig. 1B. (**B**) Cliff heights for the second experiment, after ice shelf collapse in 2065, as shown in Fig. 1C. The area shaded in red shows the parameter space for which cliff failure should occur according to the cliff failure parameterization.

We initialize the three ice sheet models and calibrate them using 2015 conditions (*12*). After initialization, we simulate a complete and instantaneous ice shelf collapse by removing all floating ice completely (red line in Fig. 1A). In reality, such removal might occur over a relatively short period, though likely not instantaneously (*10*), and could be accelerated in a warming climate through processes such as hydrofracture (*13*). However, our instantaneous removal represents a conservative estimate, which minimizes grounded thinning during ice removal and thus maximizes associated cliff heights. The cliff exposed after ice shelf collapse (Figs. 1B and 2A) is then allowed to retreat at a rate determined by the revised parameterization (*7*). To simulate a worst-case scenario, we choose the parameters that induce the highest calving rate found in that study, describing less viscous and rapidly sliding ice.

We run all three models for 100 years under constant atmospheric and oceanic forcing. In addition, the ice shelf is not allowed to regrow in any of the models and the potential stabilizing effect of sea ice, ice mélange, and icebergs on the calving rate is ignored. Including these effects would likely decrease calving and increase stability.

**Fig. 3.**
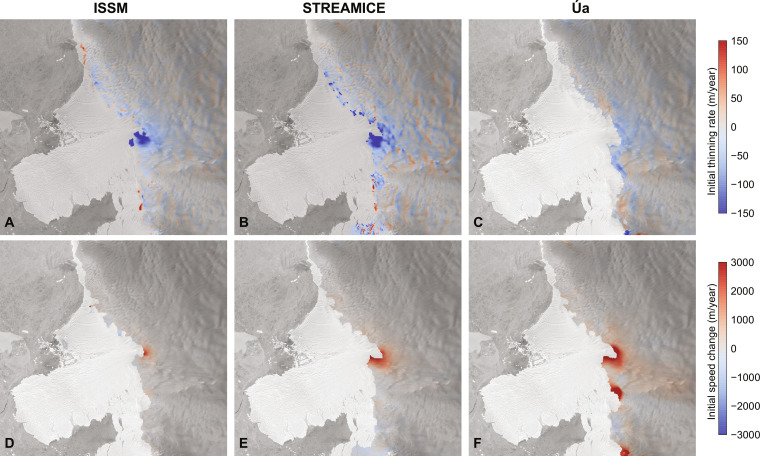
Initial modeled thinning rate and speed up after ice shelf collapse. (**A** to **C**) Initial thinning rate for the three different ice sheet models after prescribed ice shelf collapse and (**D** to **F**) associated increase in the speed of ice flow.

For all three models, the ice front remains near its initial position (Fig. 1B). The ice front does not readvance because it is not allowed to do so by design (see below). Multiple factors explain why the modeled ice front does not retreat. First, the grounding line is located on a bedrock high that is fairly shallow (∼500 m below sea level) and so the exposed ice cliff only exceeds the threshold of 135 m in a handful of locations (Fig. 2A). In these places, the calculated calving rate does not exceed 6 to 7 m/day, which is lower than the calving rate of 10 m/day used in the existing cliff failure parameterization (*4*). Furthermore, two strong negative feedbacks counteract cliff failure. (i) When the ice shelf collapses, the presence of the ice cliff leads to a strong acceleration of the ice stream (Fig. 3). All models show an instantaneous increase in flow speed of up to 3 km/year right after the initial ice shelf collapse, or a doubling of today’s ice flow speed. Ice front retreat requires that the calving rate due to cliff failure exceeds this ice speed, which is rarely the case in our simulations. (ii) This flow acceleration creates rapid ice thinning close to the ice front: we find thinning rates exceeding 150 m/year for ISSM and STREAMICE, and 100 m/year for Úa. Therefore, even if the cliff may initially retreat, the ice upstream is not necessarily thicker after a short period as was initially suggested (*4*) but can be thinner because of the high rates of dynamic thinning associated with exposing a tall ice cliff (*5*).

Contrary to previous simulations (*4*), the revised calving parameterization (*7*) is conservative and does not include subcritical ice calving processes. As a consequence, the calving rates are zero for cliff heights below 135 m, inevitably leading to an advance of all fronts with lower heights. However, in line with our worst-case scenario approach, we additionally suppress any advance of the ice front at any time throughout the computational domain. In this scenario, the calving speed is equal to ice speed where ice cliffs are below 135 m.

### Response to future ice shelf collapse

Although Thwaites Glacier may not be vulnerable to MICI today, it has been suggested that very tall (>200 m) cliffs could eventually be exposed as the grounding line continues to retreat deeper inland, as Thwaites is located in a deep submarine basin of West Antarctica. Such tall cliffs could lead to potentially rapid retreat given the strong increase in calving rate with cliff height in the revised parameterization (*7*). To test this hypothesis, we run the same ice sheet models forward in time for 50 years and induce a grounding-line retreat at a rate of 1 km/year by incrementally reducing the areal extent of basal drag at the same rate (see the Supplementary Materials) using the same constant atmospheric and oceanic forcing as assigned previously. This rate of grounding-line retreat is consistent with today’s highest rates of retreat (*14*). We use this approach to induce an extensive retreat over a brief amount of time and to reduce any potential variability between models. At the end of the 50-year simulation, we again remove the floating ice shelf entirely, as well as any areas where basal drag has been reduced following our forced groundingline retreat described above, and run the model forward with the cliff failure parameterization. The ice front is now above the limit of 135 m over large areas for all three models (Fig. 2B), as the bed topography is more than 1000 m below sea level, reaching 1400 m in a 10-km-wide trench. We only run the models for 20 years after the ice shelf collapse because if a rapid MICI-style collapse develops, it is likely to occur in the first few years of the simulation when the ice front is tallest. In other words, if MICI does not start immediately after the ice shelf collapse, it is unlikely that it will be triggered at a later stage, as illustrated by our first set of experiments. Again, while the models retreat marginally over the first couple of years of simulation after the ice shelf collapse, this retreat is arrested rapidly across all three models.

The reasons for this behavior are the same as the ones in the first set of experiments: As the grounding line retreats over the first 50 years of the simulation, the ice flow accelerates, and the ice thins considerably. Although the ice may be thick upstream of the grounding line today, by the time the grounding line retreats into the deeper basin, it also induces rapid ice thinning upstream and even if the ice shelf collapses in the future, the cliff height will likely not be as tall as previously suggested, based on today’s geometry (Fig. 2B). The two negative feedbacks of ice acceleration and ice thinning following the collapse of the ice shelf are strong enough to stop the retreat due to cliff failure, and the glacier does not retreat immediately further upstream in an uncontrollable manner, contrary to what is expected under MICI. This behavior is identical to that identified by (*5*), where retreat is stabilized by thinning. The simulations conducted here show that rapid acceleration upstream further reduces the tendency to retreat.

## DISCUSSION

It is important to note that we chose “worst-case scenarios” for all parameters in these experiments. First, we took the largest calving rates proposed in the revised parameterization (*7*). Second, in both sets of experiments, we applied a complete and instantaneous collapse of Thwaites’ ice shelf, which exposes a taller cliff than if the ice shelf were too slowly disintegrate, as a gradual disintegration would induce ice acceleration and thinning (*15*). We also do not allow the front to advance once the ice shelves disintegrate. Last, we assumed that, as the ice calves off, it is transported by the ocean and the granular media in front of the calving face do not exert any buttressing or slow down calving, which is a mechanism that has been observed in Greenland (*16*).

The revised parameterization of cliff calving (*7*), however, is based on sequential modeling of viscous and brittle processes, in which oversteepening of the frontal cliff is followed by tensile failure. The calving rate law was based on the waiting time for sufficient oversteepening to occur and the magnitude of the ensuing calving event. However, this model likely produces lower calving rates—and a higher cliff height failure threshold—than a more physically realistic scenario in which viscous and brittle shear deformation and tensile failure can interact continuously. In view of this, Crawford *et al*. (*7*) argued that their cliff calving rate function, including the height threshold at which cliff failure initiates, should be regarded as conservative.

Our study omits more conventional calving processes, such as the release of tabular icebergs, which may produce rapid calving in areas where longitudinal stretching rates are high. This omission may contribute to an unrealistic “gap” in calving losses below the 135-m threshold for cliff failure. Nevertheless, contrasting our simulations to previous work (*4*) shows that the onset of MICI at Thwaites Glacier within the coming decades is much less likely than previously argued. Additional experiments (see the Supplementary Materials) show that the calving rate would need to increase by a factor of 25 to trigger an unstoppable retreat in the region.

Our results show that the Amundsen Sea Embayment, and so the West Antarctic Ice Sheet, is not vulnerable to MICI under likely 21st-century ice configurations based on a revised, more physically motivated parameterization of cliff failure. This result is robust across three different ice sheet models. However, our results do not suggest that the West Antarctic Ice Sheet is stable. It has been shown that Thwaites Glacier is potentially subject to marine ice sheet instability (MISI), a feedback process involving grounding-line retreat into deeper-bedded areas (*17*), and which can lead to high rates of sea-level rise over several centuries. Our experiments do not preclude MISI unfolding in the future for the West Antarctic Ice Sheet; rather, we argue that the hypothetical process of MICI may not play a role in its demise in the 21st century. With the conservative cliff failure parameterization implemented in our study, the calving rate would need to be 25 times higher to trigger a retreat of the calving front after the collapse of its ice shelf. The high rates of sea level rise that have been suggested in previous studies and in the IPCC remain speculative. Given the contrasting results between the limited number of modeling studies that represent cliff failure, with varying implementation and different climate forcing, there is a pressing need for further investigations into the processes underpinning cliff failure. A better understanding of the processes and parameters that control the critical height and rates of calving rates near the onset of failure where current parameterizations diverge is especially needed.

## MATERIALS AND METHODS

The ice sheet models used in this study are the Ice-sheet and Sea-level System Model [ISSM (*18*)], Úa, and STREAMICE, which is a package of the MITgcm (*19*). The initialization procedure of the models is similar to the one described in ample detail in (*12*). We only highlight key elements here. ISSM and Úa use here the two-dimensional depth-integrated shallow shelf approximation (*20*). ISSM’s model comprises 75,000 elements with a resolution of 1.5 km in fast-moving regions, gradually increasing closer to the divides. ISSM uses a subelement grounding-line parameterization (*21*) and a level-set approach to model the motion of the calving front (*22*, *23*). Úa uses a mesh initially consisting of 90,000 elements, with a resolution of 1 km at the grounding lines and calving fronts, increasing toward 10 km upstream, and also models the dynamics of the ice front based on the level-set method. STREAMICE (*24*) uses a hybrid stress balance (*25*) on a rectangular grid using a combination of finite-element and finite-volume methods. In this study, STREAMICE has a resolution of 1 km at the grounding line, expanding to ∼5 km at the boundary of the domain. To evolve the calving front, a flux-based method (*26*) is adopted, which maintains “partial” cells oceanward of the calving front which do not play a role in the momentum balance until filled. All models use a Weertman sliding law (*27*) and use data assimilation methods for initialization. See the Supplementary Materials for more information on model initialization and modeling protocol.

## Supplementary Materials

### This PDF file includes:

Supplementary Text Fig. S1 References
