# Supplementary material for: The West Antarctic Ice Sheet may not be vulnerable to marine ice cliff instability during the 21st century
Source: Sci Adv. 2024 Aug 21;10(34):eado7794. doi: 10.1126/sciadv.ado7794 (PMC11338217; doi:10.1126/sciadv.ado7794)
Supplement: Supplementary file 1 — Supplementary Text Fig. S1 References [file sciadv.ado7794_sm.pdf]

Science Advances
AAAS

Supplementary Materials for

The West Antarctic Ice Sheet may not be vulnerable to marine ice cliff instability during the 21st century

Mathieu Morlighem *et al.*

Corresponding author: Mathieu Morlighem, mathieu.morlighem@dartmouth.edu

Sci. Adv. **10**, eado7794 (2024)
DOI: 10.1126/sciadv.ado7794

The PDF file includes:

Supplementary Text
Fig. S1
References

1 Boundary conditions and initial state

While several sliding laws have been tested, leading to similar results, the models shown in the main text are all based on a Weertmann sliding law [27] with an exponent $m = 3$:

$$\tau_b = C^2 v_b^{1/m} \tag{1}$$

where τ_b is the magnitude of the basal stress and v_b is the sliding speed. The sliding law coefficient, C, is calibrated using surface velocities from satellite interferometry [28]. Úa and STREAMICE also inverted for the rate factor, A, while ISSM relied on a temperature based relationship using the results of a thermal model.

All models use an initial geometry from BedMachine Antarctica v3 [29], and the models are then run forward under constant climatic forcings. The surface mass balance is from RACMO2.3, integrated between 1979 and 2011, and basal melt under floating ice is parameterized using a depth-relationship. The melt rate linearly increases from 0 at the surface of the ocean to 50 m/yr at a depth of 500 m.

2 Experiments

2.1 Calving law

Here, we employ the calving law from [6], which reads:

$$c = I\ H_c^{\alpha} \tag{2}$$

where c is the calving rate (in m/day), H_c is the cliff height (in m), and I and α are constants (SI). We choose the highest calving rate of their paper with $\alpha = 7.3$ and I =1.9e-16 SI, which is find for warm (-5°) sliding ice. The ISSM model for InitMIP suggest a depth-average temperature of ~ -10°C in this sector [30], which would lead to a lower calving rate.

The calving law only applies when the cliff height is greater than 135 m. When ice cliffs are less than 135 m high, or when the calving rate is smaller than the ice speed, we assume that the calving rate is exactly equal to the ice velocity, $c = v$, so that the terminus does not readvance and the ice shelf is not allowed to regrow.

2.2 Ice shelf collapse

In the first set of experiments, we remove all floating components of the models, exposing tall grounded cliffs at today's grounding lines. We then run the models forward for 100 years using the forcings described above.

2.3 Future Ice shelf collapse

The idea of this experiment is that even if complete ice shelf removal today does not result in catastrophic collapse due to cliff failure, we investigate if it could in the future, when the grounding line has retreated to deeper bed. We impose a "retreat" of basal friction (C) that effectively moves the extent of basal traction inland at a rate of 1 km/yr within Thwaites' basin, and use this retreat as a forcing in a time-dependent run with the above melt and SMB forcing, but no change in calving front position (i.e., the ice shelf is preserved until the end of this initial simulation). Basal drag removal was implemented by generating successive masks based on a signed distance function; within these masks, C was set to zero.

We then take the geometry after 50 years of simulation, and remove all floating ice and also all ice with signed distance from the original grounding line under 50 km within the Thwaites catchment. Once this new geometry is generated, we run a time-dependent calving experiment

3 Sensitivity analysis

In order to check the sensitivity of the cliff-failure parameterization to ice thickness, we test a scaling approach in which equation 2 is rescaled as follows:

$$c = I \left(H_c \frac{H_{135}}{H_0} \right)^{\alpha} \tag{3}$$

where $H_{135} = 135$ m is the original cliff threshold in [6], and H_0 is a lower threshold cliff height. This equation can be rewritten as:

$$c = \left(\frac{H_{135}}{H_0} \right)^{\alpha} I \ (H_c)^{\alpha} \tag{4}$$

For example, if we lower the cliff height to 110 m, this calving rate will be $(135/110)^{7.3} = 4.46$ times higher than the original calving rate. We run some of these experiments with ISSM to test how resilient the ice front would be for

potentially lower threshold cliff heights. In order to start a rapid retreat, we need to choose $H_0 = 80$ m for Thwaites Glacier, and $H_0 = 90$ m for Kohler Glacier, which involves multiplying the initial parameterization by a factor of 45 and 25 respectively (See figure S1).

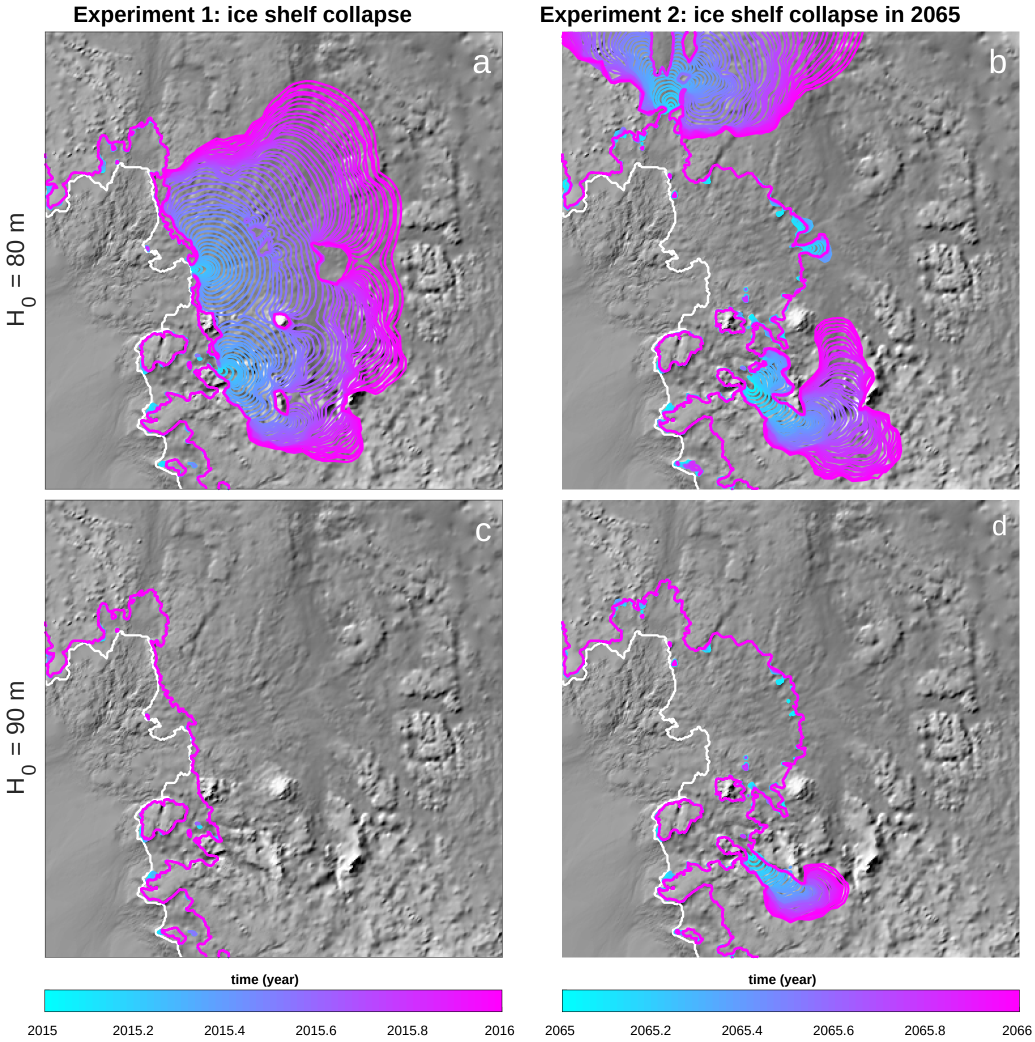

Figure S1: Ice front positions over the first year of simulation for H_0=80 m (a,b) and H_0=90 m (c,d) for the two experiments described in the manuscript.

REFERENCES AND NOTES

1. Intergovernmental Panel on Climate Change, *Climate Change 2021: The Physical Science Basis. Contribution of Working Group I to the Sixth Assessment Report of the Intergovernmental Panel on Climate Change* (Cambridge Univ. Press, 2021).

2. J. N. Bassis, C. C. Walker, Upper and lower limits on the stability of calving glaciers from the yield strength envelope of ice. *Proc. R. Soc. A: Math. Phys. Eng. Sci.* **468**, 913–931 (2012).

3. D. Pollard, R. M. DeConto, R. B. Alley, Potential Antarctic Ice Sheet retreat driven by hydrofracturing and ice cliff failure. *Earth Planet. Sci. Lett.* **412**, 112–121 (2015).

4. R. M. DeConto, D. Pollard, Contribution of Antarctica to past and future sea-level rise. *Nature* **531**, 591–597 (2016).

5. J. N. Bassis, B. Berg, A. J. Crawford, D. I. Benn, Transition to marine ice cliff instability controlled by ice thickness gradients and velocity. *Science* **372**, 1342–1344 (2021).

6. R. M. DeConto, D. Pollard, R. B. Alley, I. Velicogna, E. Gasson, N. Gomez, S. Sadai, A. Contron, D. M. Gilford, E. L. Ashe, R. E. Kopp, D. Li, A. Dutton, The Paris Climate Agreement and future sea-level rise from Antarctica. *Nature* **593**, 83–89 (2021).

7. A. J. Crawford, D. I. Benn, J. Todd, J. A. Astrom, J. N. Bassis, T. Zwinger, Marine ice-cliff instability modeling shows mixed-mode ice-cliff failure and yields calving rate parameterization. *Nat. Commun.* **12**, 2701 (2021).

8. H. Seroussi, V. Verjans, S. Nowicki, A. J. Payne, H. Goelzer, W. H. Lipscomb, A. Abe-Ouchi, C. Agosta, T. Albrecht, X. Asay-Davis, A. Barthel, R. Calov, R. Cullather, C. Dumas, B. K. Galton-Fenzi, R. Gladstone, N. R. Golledge, J. M. Gregory, R. Greve, T. Hattermann, M. J. Hoffman, A. Humbert, P. Huybrechts, N. C. Jourdain, T. Kleiner, E. Larour, G. R. Leguy, D. P. Lowry, C. M. Little, M. Morlighem, F. Pattyn, T. Pelle, S. F. Price, A. Quiquet, R. Reese, N.-J.

Schlegel, A. Shepherd, E. Simon, R. S. Smith, F. Straneo, S. Sun, L. D. Trusel, J. Van Breedam, P. Van Katwyk, R. S. W. van de Wal, R. Winkelmann, C. Zhao, T. Zhang, T. Zwinger, Insights into the vulnerability of Antarctic glaciers from the ISMIP6 ice sheet model ensemble and associated uncertainty. *Cryosphere* **17**, 5197–5217 (2023).

9. B. W. J. Miles, C. R. Stokes, A. Jenkins, J. R. Jordan, S. S. R. Jamieson, G. H. Gudmundsson, Intermittent structural weakening and acceleration of the Thwaites Glacier Tongue between 2000 and 2018. *J. Glaciol.* **66**, 485–495 (2020).

10. K. E. Alley, C. T. Wild, A. Luckman, T. A. Scambos, M. Truffer, E. C. Pettit, A. Muto, B. Wallin, M. Klinger, T. Sutterley, S. F. Child, C. Hulen, J. T. M. Lenaerts, M. Maclennan, E. Keenan, D. Dunmire, Two decades of dynamic change and progressive destabilization on the Thwaites Eastern Ice Shelf. *Cryosphere* **15**, 5187–5203 (2021).

11. D. I. Benn, A. Luckman, J. A. Åström, A. J. Crawford, S. L. Cornford, S. L. Bevan, T. Zwinger, R. Gladstone, K. Alley, E. Pettit, J. Bassis, Rapid fragmentation of thwaites eastern ice shelf. *Cryosphere* **16**, 2545–2564 (2022).

12. J. M. Barnes, T. D. dos Santos, D. Goldberg, G. H. Gudmundsson, M. Morlighem, J. De Rydt, The transferability of adjoint inversion products between different ice flow models. *Cryosphere* **15**, 1975–2000 (2021).

13. C. Y. Lai, J. Kingslake, M. G. Wearing, P.-H. C. Chen, P. Gentine, J. Li, J. J. Spergel, J. M. van Wessem, Vulnerability of Antarctica's ice shelves to meltwater-driven fracture. *Nature* **584**, 574–578 (2020).

14. P. Milillo, E. Rignot, P. Rizzoli, B. Scheuchl, J. Mouginot, J. Bueso-Bello, P. Prats-Iraola, Heterogeneous retreat and ice melt of Thwaites Glacier, West Antarctica, *Sci. Adv.* **5**, eaau3433 (2019).

15. F. Clerc, B. M. Minchew, M. D. Behn, Marine ice cliff instability mitigated by slow removal of ice shelves. *Geophys. Res. Lett.* **46**, 12108–12116 (2019).

16. J. M. Amundson, M. Fahnestock, M. Truffer, J. Brown, M. P. Lüthi, R. J. Motyka, Ice mélange dynamics and implications for terminus stability, Jakobshavn Isbræ, Greenland. *J. Geophys. Res. - Earth Surface* **115**, doi.org/10.1029/2009JF001405 (2010).

17. I. Joughin, B. E. Smith, B. Medley, Marine ice sheet collapse potentially under way for the Thwaites Glacier Basin, West Antarctica. *Science* **344**, 735–738 (2014).

18. E. Larour, H. Seroussi, M. Morlighem, E. Rignot, Continental scale, high order, high spatial resolution, ice sheet modeling using the Ice Sheet System Model (ISSM). *J. Geophys. Res.* **117**, doi.org/10.1029/2011JF002140 (2012).

19. MITgcm Group, "MITgcm release 2 manual" (Tech. Rep., MIT/EAPS, Cambridge, MA, 2016).

20. D. R. MacAyeal, Ice-shelf response to ice-stream discharge fluctuations: III. The effects of ice-stream imbalance on the ross ice shelf, Antarctica. *J. Glaciol.* **35**, 38–42 (1989).

21. H. Seroussi, M. Morlighem, E. Larour, E. Rignot, A. Khazendar, Hydrostatic grounding line parameterization in ice sheet models. *Cryosphere*, **8**, 2075–2087 (2014).

22. J. H. Bondzio, H. Seroussi, M. Morlighem, T. Kleiner, M. Rückamp, A. Humbert, E. Larour, Modelling calving front dynamics using a level-set method: Application to Jakobshavn Isbræ, West Greenland. *Cryosphere* **10**, 497–510 (2016).

23. M. Morlighem, J. Bondzio, H. Seroussi, E. Rignot, E. Larour, A. Humbert, S.-A. Rebuffi, Modeling of Store Gletscher's calving dynamics, West Greenland, in response to ocean thermal forcing. *Geophys. Res. Lett.* **43**, 2659–2666 (2016).

24. D. N. Goldberg, P. Heimbach, Parameter and state estimation with a time-dependent adjoint marine ice sheet model. *Cryosphere* **7**, 1659–1678 (2013).

25. D. N. Goldberg, A variationally derived, depth-integrated approximation to a higher-order glaciological flow model. *J. Glaciol.* **57**, 157–170 (2011).

26. T. Albrecht, M. Martin, M. Haseloff, R. Winkelmann, A. Levermann, Parameterization for subgrid-scale motion of ice-shelf calving fronts. *Cryosphere* **5**, 35–44 (2011).

27. J. Weertman, On the sliding of glaciers. *J. Glaciol.* **3**, 33–38 (1957).

28. J. Mouginot, E. Rignot, B. Scheuchl, R. Millan, Comprehensive annual ice sheet velocity mapping using Landsat-8, Sentinel-1, and RADARSAT-2 data. *Remote Sens.* **9**, 364 (2017).

29. M. Morlighem, E. Rignot, T. Binder, D. Blankenship, R. Drews, G. Eagles, O. Eisen, F. Ferraccioli, R. Forsberg, P. Fretwell, V. Goel, J. S. Greenbaum, H. Gudmundsson, J. Guo, V. Helm, C. Hofstede, I. Howat, A. Humbert, W. Jokat, N. B. Karlsson, W. S. Lee, K. Matsuoka, R. Millan, J. Mouginot, J. Paden, F. Pattyn, J. Roberts, S. Rosier, A. Ruppel, H. Seroussi, E. C. Smith, D. Steinhage, B. Sun, Michiel R. van den Broeke, T. D. van Ommen, M. van Wessem, D. A. Young, Deep glacial troughs and stabilizing ridges unveiled beneath the margins of the Antarctic ice sheet. *Nat. Geosci.* **13**, 132–137 (2020).

30. H. Seroussi, S. Nowicki, E. Simon, A. Abe-Ouchi, T. Albrecht, J. Brondex, S. Cornford, C. Dumas, F. Gillet-Chaulet, H. Goelzer, N. R. Golledge, J. M. Gregory, R. Greve, M. J. Hoffman, A. Humbert, P. Huybrechts, T. Kleiner, E. Larour, G. Leguy, W. H. Lipscomb, D. Lowry, M. Mengel, M. Morlighem, F. Pattyn, A. J. Payne, D. Pollard, S. F. Price, A. Quiquet, T. J. Reerink, R. Reese, C. B. Rodehacke, N.-J. Schlegel, A. Shepherd, S. Sun, J. Sutter, J. Van Breedam, R. S. W. van de Wal, R. Winkelmann, T. Zhang, initMIP-Antarctica: An ice sheet model initialization experiment of ISMIP6. *Cryosphere* **13**, 1441–1471 (2019).
